# CHARACTERISTICS ASSOCIATED WITH PRIMARY CARE–BASED DEMENTIA SCREENING VISIT COMPLETION IN THE ERADAR STUDY

**DOI:** 10.1093/geroni/igae098.1129

**Published:** 2024-12-31

**Authors:** Leah Karliner, Sascha Dublin, Yates Coley, Clarissa Hsu, Tyler Barrett, Deborah Barnes

**Affiliations:** University of California San Francisco, San Francisco, California, United States; Kaiser Permanente Washington Health Research Institute, Seattle, Washington, United States; Kaiser Permanente Washington Health Research Institute, Seattle, Washington, United States; Kaiser Permanente Washington, Seattle, Washington, United States; Kaiser Permanente Washington Health Research Institute, Seattle, Washington, United States; University of California San Francisco, San Francisco, California, United States

## Abstract

Nearly 7 million people in the U.S. are living with dementia, but approximately half are undiagnosed. We are performing a randomized, controlled trial in two healthcare systems (Kaiser Permanent Washington-KPWA, University of California San Francisco-UCSF) of targeted dementia screening. Among primary care patients ≥ age 65, high-risk individuals are identified using a predictive algorithm, eRADAR (electronic health record Risk of Alzheimer’s and Dementia Assessment Rule), and invited for a “brain health” screening visit (BHV). Among 1709 KPWA patients in the intervention group, 506 (30%) completed a BHV; ongoing UCSF recruitment is similar (N=68/221; 31%). Visit completion rates were lower for participants of color (Asian/Black/Latinx) at 18% (N=51/336) compared with non-Hispanic whites at 34% (N=446/1,313), adjusted OR 0.36, 95% CI 0.26-0.49. Completion rates were 33% (N=30/90) among people aged 65-74 vs. 16% (N=44/268) for those aged 90+, adjusted OR per decade 0.71, 95% CI 0.56-0.90. Prior diagnoses of memory problems/concerns or mild cognitive impairment (MCI) were associated with higher odds of completion (adjusted OR 1.49; CI 1.13-1.95), and so was lower comorbidity score (adjusted OR 1.01; CI 1.00-1.03). eRADAR score and gender were not associated with BHV completion. The eRADAR study participation rate is similar to other dementia screening studies. Younger, white patients and those who might be aware of their cognitive deficits–as demonstrated by prior diagnoses–appear more likely to participate. As disease-modifying treatments increasingly become available, further research with communities of color to understand facilitators and barriers to engagement in primary-care dementia assessment is vital to prevent care disparities.

